# Salivary chemokines and growth factors in patients with ischemic stroke

**DOI:** 10.1038/s41598-025-97974-5

**Published:** 2025-04-12

**Authors:** Dominika Forszt, Karolina Gerreth, Kamila Karpienko, Anna Zalewska, Katarzyna Hojan, Renata Marchewka, Marzena Bielas, Mateusz Maciejczyk

**Affiliations:** 1 Department of Risk Group Dentistry, Chair of Pediatric Dentistry, Poznan University of Medical Sciences, Poznan, Poland; 2 Students Scientific Club “Biochemistry of Civilization Diseases” at the Department of Hygiene, Epidemiology and Ergonomics, Medical University of Bialystok, Bialystok, Poland; 3 Experimental Dentistry Laboratory, Department of Conservative Dentistry, Medical University of Bialystok, Bialystok, Poland; 4 Department of Occupational Therapy, Poznan University of Medical Sciences, Poznan, Poland; 5 Department of Rehabilitation, Greater Poland Cancer Centre, Poznan, Poland; 6 Neurorehabilitation Ward, Greater Poland Provincial Hospital, 60-480 Poznan, Poland; 7 Department of Family Medicine, Poznan University of Medical Sciences, Poznan, Poland; 8 Department of Hygiene, Epidemiology and Ergonomics, Medical University of Bialystok, Mickiewicza 2C Street, 15-089 Bialystok, Poland

**Keywords:** Stroke, Saliva, Chemokines, Growth factors, Biomarkers, Salivary diagnostics, Diagnostic markers, Neurology

## Abstract

Stroke is a serious health problem that affects an increasing number of people. As a result of the blockage of blood flow, tissue necrosis occurs in areas of the brain supplied by the damaged vessel, and leads to the development of inflammation. Changes that occur in the brain allow molecules to enter the blood, and it has been suggested that some can also penetrate the saliva. This study is the first to assess the profile of 25 chemokines and growth factors in the saliva of stroke survivors compared to a control group. 22 stroke survivors and 22 individuals matched by age and gender were enrolled in the study. Salivary chemokines and growth factors were assessed using the multiplex ELISA method. In the unstimulated saliva of stroke patients, we demonstrated significantly higher levels of chemotactic factors (CTACK/CCL27, IL-8/CXCL8, MIG/CXCL9, MIF) and growth factors (basic FGF, G-CSF, HGF, LIF, VEGF) compared to controls. The levels of MCP-3/CCL7, eotaxin/CCL11, IP-10/CXCL10, IL-3/MCGF, and PDGF-BB were lower in the saliva of the study group. The concentration of basic FGF negatively correlated with cognitive function as measured by the Addenbrooke’s Cognitive Examination (ACE) scale (*p* = 0.007 *r* = − 0.56), while salivary IL-3 and LIF levels positively correlated with scores on the Functional Independence Measure (FIM) scale (*p* = 0.019 *r* = 0.53; *p*  = 0.033 *r* = 0.47, respectively). Receiver Operating Characteristic (ROC) analysis showed that salivary basic FGF, HGF, IL-3 and LIF can distinguish ischemic stroke patients from the control group with high sensitivity and specificity. In conclusion, disruptions in chemokine and growth factor levels in saliva may suggest an inflammatory etiology of ischemic stroke. Salivary basic FGF, HGF, IL-3 and LIF could serve as potential biomarkers for stroke. Further research is needed to illuminate the differences in salivary inflammatory mediator profiles in stroke and to evaluate the diagnostic utility of chemokines and growth factors in clinical practice.

**Supplementary Information:**

The online version contains supplementary material available at 10.1038/s41598-025-97974-5.

## Introduction

Stroke is a major public health issue. With a 70% increase in the incidence of strokes between 1990 and 2019^1^, it’s one of the leading causes of death worldwide. In total, 1,802,560 stroke incidents were recorded in 2019, of which 70% were ischemic. The epidemiology of stroke is influenced by a complex interaction of environmental, genetic, and lifestyle factors. Commonly cited risk factors include hypertension, smoking, diabetes, obesity, and heart disease^2^.

Physiologically, the brain consumes approximately 20% of the oxygen supplied to the body^3^. The lack of blood supply in ischemic stroke leads to insufficient delivery of nutrients and oxygen to brain tissue, resulting in neuronal death^4^. Neuronal bioenergetic disturbances promote mitochondrial damage-associated molecular patterns (DAMPs), leading to the development of an inflammatory response^5,6^. Neuroinflammation is characterized by microglial activation, leukocyte infiltration, inflammatory cell aggregation, and subsequent release and further recruitment of cytokines, chemokines, and various neurotoxic molecules^2^. The pro-inflammatory pathway weakens the blood-brain barrier (BBB) and causes vascular “leakage” of inflammatory mediators^7^.

Chemokines play a key role in regulating the immune response and inflammation in stroke patients^2^. The secretion of chemokines enhances the transport of immune cells across the BBB into the brain^7^. Depending on the position of the two N-terminal cysteine residues, chemokines are divided into two subfamilies: CXC (where the first two cysteine residues are separated by an amino acid) and CC (where the cysteines are adjacent to each other)^8^. The structural classification of chemokines indicates differences in their selectivity for target cells, with the CXC subfamily attracting neutrophils and the CC subfamily affecting monocytes, macrophages, and lymphocytes^9^. Infiltrating inflammatory cells damage the ischemic brain by synthesizing various cytotoxic mediators (nitric oxide (NO), free radicals, and prostanoids), which prolong the inflammatory response^10^. Therefore, chemokines contribute to BBB damage^11^. On the other hand, they also participate in other nervous system functions, such as angiogenesis and neuronal survival^12^.

Growth factors are polypeptides or proteins released by many types of cells^13^. The pleiotropic effects of growth factors include neuroprotection, stimulation of stem cells, promotion of angiogenesis and neurogenesis, as well as anti-apoptotic and anti-inflammatory properties^14^. The neuroprotective effect of growth factors results from their ability to mitigate the negative consequences of stroke. Indeed, increased production of growth factors such as VEGF (vascular endothelial growth factor), G-CSF (granulocyte colony-stimulating factor), and SDF-1α/CCL12 (stromal cell-derived factor 1/chemokine (C-X-C motif) ligand 12) in patients with ischemic stroke has been correlated with favorable functional outcomes and a reduction in the extent of pathological changes in the brain^15,16^.

Neuroradiological techniques (CT and MRI) are essential elements in stroke diagnostics. However, there is currently a lack of inexpensive, widely available, and sensitive diagnostic tests for early laboratory diagnosis, as well as effective treatment of stroke patients. Chemokines and growth factors have a significant impact on the course of stroke, making their potential use in stroke diagnostics a topic of interest. Previous studies have shown that the concentrations of IL-8/CXCL8 (interleukin 8/chemokine (C-X-C motif) ligand 8)^17^, MIF (macrophage migration inhibitory factor)^18^, G-CSF^19^, HGF (hepatocyte growth factor)^20^ and VEGF^21^ were significantly higher in the blood of stroke patients and correlated with disease progression. However, these markers have not been measured in the saliva of stroke patients. Saliva as a diagnostic material is gaining increasing attention due to the comfort and non-invasive nature of sample collection^22^. Saliva collection reduces patient anxiety and allows for multiple tests to be conducted throughout the day^23^. Another advantage of saliva is its greater safety compared to blood, as it minimizes the risk of HIV and hepatitis infections^23^. Therefore, it is not surprising that there is a growing number of studies indicating the presence of various biomarkers in saliva, including stroke biomarkers^24–31^.

Due to the increasing number of stroke cases and the lack of non-invasive and easily accessible diagnostic methods, this study is the first to assess the profile of 25 chemokines and growth factors in the unstimulated saliva of patients with ischemic stroke. For this purpose, we used multiplex ELISA technology, which allows for the simultaneous analysis of multiple proteins in a small volume sample. A comprehensive cytokine profile of saliva could serve as a solid basis for the development of non-invasive stroke biomarkers.

## Materials and methods

### Bioethics committee

The research was approved by the Bioethics Committee of the Poznan University of Medical Sciences (59/19, 890/19, 504/21) adhering to the principles of the Declaration of Helsinki on human experimentations. Each patient signed an informed consent form after receiving detailed information about the purpose and scope of the study.

### Participants

#### Study group

Participation in the study was voluntary, and written consent was obtained from each individual. The study group consisted of patients admitted to the Neurorehabilitation Ward at the Greater Poland Provincial Hospital (Kiekrz, Poland), where they were undergoing recuperation following neurological disorders. Patients were recruited within the first two days after admission to the ward (between the 10th and 13th day after the stroke). Patients were classified according to the inclusion and exclusion criteria presented in Table 1.

**Table 1:** Inclusion and exclusion criteria.

Inclusion criteria	Exclusion criteria
Individuals over 18 years old	Individuals under 18 years old
Radiologically confirmed ischemic stroke	Lack of radiological confirmation of ischemic stroke
First-time stroke	Second or subsequent stroke
Ability to provide informed consent for participation in the study and for saliva collection	Inability to provide informed consent for the study and saliva sampling
Ability to provide a saliva sample	Inability to provide a saliva sample
Individuals who were not suffering from autoimmune, psychiatric, and/or thromboembolic diseases, such as limb thrombosis, pulmonary embolism, or recent acute coronary syndrome	Individuals suffering from autoimmune, psychiatric, and/or thromboembolic diseases, such aslimb thrombosis, pulmonary embolism, or recent acute coronary syndrome
Patients who did not experience post-stroke infections	Patients with post-stroke infections
Subjects without a history of salivary gland diseases	Subjects with salivary gland diseases
No cigarette smoking	Cigarette smoking
Stroke that was not treated with thrombectomy or thrombolysis	Stroke treated with thrombectomy or thrombolysis

**Fig. 1:**
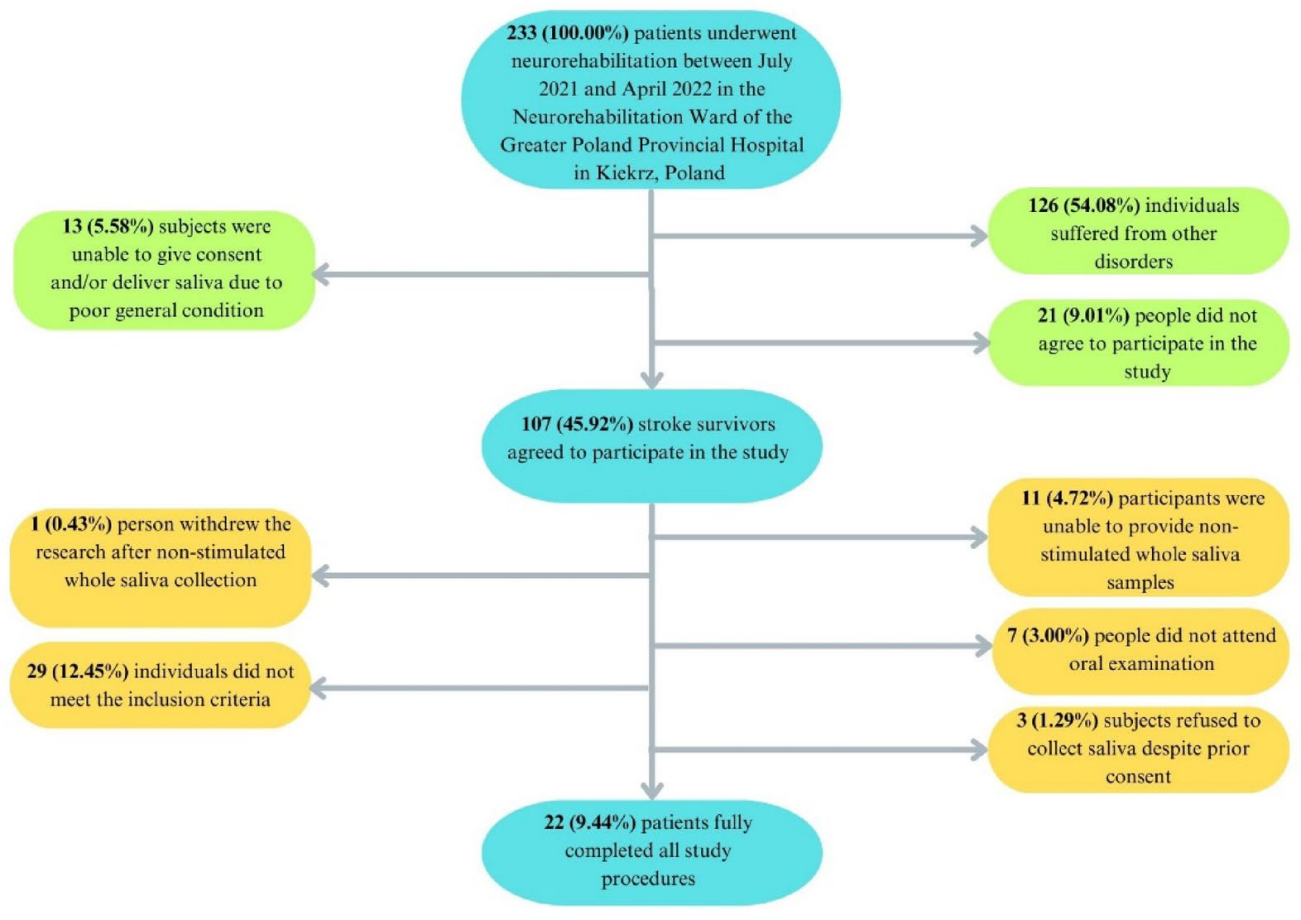
Patient selection flow chart.

After applying the inclusion and exclusion criteria, out of 233 participants, 22 patients with ischemic stroke were selected for the study (Fig. 1).

### Control group

Twenty-two individuals were recruited into the control group. Participants were matched by sex, age, oral hygiene, dental status, and periodontal condition to correspond with the study group. Volunteers were recruited during a follow-up visit to the Department of Conservative Dentistry at the Medical University of Bialystok (Bialystok, Poland). The study involved adults over the age of 18 who were able to provide informed consent and saliva samples. The exclusion criteria included being under 18 years of age, cigarette smoking, and the inability to provide informed consent or saliva samples. Before the study commenced, each person presented a medical certificate confirming their good health. A detailed patient history was taken from each participant in order to gather information about chronic diseases, education level, smoking habits, and lifestyle. Control group participants were instructed on generally accepted physical activity recommendations. Additionally, participants followed a standard, unrestricted diet.

### Research procedures

The procedures included saliva testing, assessment of oral health, and evaluation of the impact of stroke on the functional status of the patients.

#### Saliva testing

Before obtaining saliva samples, participants from both the study and control groups were instructed to refrain from eating and drinking, intense physical activity, as well as oral hygiene practices for 8 h before the collection. The procedures were conducted between 8:00 and 10:00 in the morning. To collect saliva samples, patients were invited to a secluded room and given 5 min to acclimate to their surroundings before starting the procedure. Participants were then asked to clean their mouths to minimize the risk of sample contamination. Therefore, each participant was instructed to rinse their mouth twice with distilled water, which was at room temperature. The spitting method was chosen for saliva collection. To ensure that the procedure was done correctly, the physician explained the method to the participants. The patient was instructed to lean forward and spit into a calibrated tube for 10 min. All tubes were sterile, and the patient held a cup with ice in which the tube was placed. The supernatant obtained after centrifuging the samples was used for the study. Centrifugation was performed at a temperature of + 4 °C, at a speed of 3000×g, which lasted for 20 min. The resulting supernatant was stored at − 80 °C^32,33^.

Subsequently, the concentration of biomarkers in saliva was evaluated. The specific biomarkers are presented in Table 2, and the Bio-Plex Pro Human Cytokine Assay (Bio-Rad Laboratories, Inc., Hercules, CA, USA) was used for their assessment. The Bio-Plex technology involves binding of the investigated markers to specific antibodies attached to magnetic beads. To initiate this reaction, the supernatant was mixed with the magnetic beads. After binding the targeted markers, the unbound molecules were removed through a series of washes, and a sandwich compound was created by introducing a biotinylated detection antibody. The final complex was formed by adding a streptavidin-phycoerythrin (SA-PE) conjugate. Results were read using the dedicated plate reader Bio-Plex 200 (Bio-Rad Laboratories, Inc., Hercules, CA, USA). Prior to the assays, the reader was validated and calibrated to ensure the reliability and consistency of the results using the commercially available Bio-Plex^®^ Validation Kit 4.0 and Bio-Plex Calibration Kit (BIO-RAD, Hercules, CA, USA).

**Table 2:** Salivary chemokines and growth factors in stroke patients.

Salivary chemokines	Salivary growth factors
• MCP-1/CCL2: monocyte chemoattractant protein-1/chemokine ligands 2; • MIP-1α/CCL3: macrophage inflammatory protein 1alpha/ chemokine ligands 3; • MIP-1β/CCL4: macrophage inflammatory protein 1 beta/ chemokine ligands 4; • RANTES/CCL5: regulated on activation, normal T-cell expressed and secreted/chemokine ligand 5; • MCP-3/CCL7: monocyte chemoattractant protein-3/chemokine ligands 7; • Eotaxin/CCL11: eotaxin/chemokine ligand 11; • CTACK/CCL27: cutaneous T-cell-attracting chemokine/chemokine ligands 27; • GRO-α/CXCL1: growth-regulated alpha protein/chemokine (C-X-C motif) ligand 1; • IL-8/CXCL8: interleukin 8/chemokine (C-X-C motif) ligand 8; • MIG/CXCL9: monokine induced by gamma interferon/chemokine (C-X-C motif) ligand 9; • IP-10/CXCL10: interferon gamma-induced protein 10/chemokine (C-X-C motif) ligand 10; • SDF-1α/CXCL12: stromal cell-derived factor 1/chemokine (C-X-C motif) ligand 12; • MIF: macrophage migration inhibitory factor	• Basic FGF; fibroblast growth factor; • G-CSF: granulocyte colony-stimulating factor, • HGF: hepatocyte growth factor; • IL-3/MCGF: interleukin-3/ mast cell growth factor; • LIF: leukemia inhibitory factor; • M-CSF: macrophage colony-stimulating factor; • PDGF-BB; platelet-derived growth factor isoform BB; • VEGF: vascular endothelial growth factor; • GM-CSF: granulocyte-macrophage colony-stimulating factor; • SCGF-β: stem cell growth factor-beta; • SCF: stem cell factor; • NGF-β: nerve growth factor-β

Since the concentration of biomarkers in saliva depends on the secretory activity of the salivary glands, their levels were standardized to total protein content, which was determined using the bicinchoninic acid (BCA) method (Thermo Scientific PIERCE BCA Protein Assay kit (Rockford, IL, USA).

####  Oral health assessment

After saliva collection, an oral health assessment was performed using the DMFT (Decayed, missing, and filled permanent teeth), PlI (Plaque), and GI (Gingival) indices^34^.

DMFT enabled the evaluation of the presence of dental caries by summing up all the teeth with carious lesions (DT) and past consequences related to their presence, i.e., the number of teeth filled due to caries (FT) and removed due to caries (MT).

PlI was used to assess oral hygiene according to the following criteria:

- 0 – no plaque present,
- 1 – plaque visible only after probing,
- 2 – visible to the naked eye, moderate plaque accumulation,
- 3 – significant plaque accumulation.

GI was used to assess gingival condition based on the following scale:

- 0 – no gingival inflammation,
- 1 – redness and swelling of the gums,
- 2 – redness, swelling of the gums, and bleeding on probing,
- 3 – redness, swelling of the gums, and self-reported spontaneous gingival bleeding^34^.

Before beginning the oral health assessment, an experienced specialist in dentistry (A.Z.) conducted training and calibration of two dentists (D.F. and K.G.). The intra-examiner and inter-examiner agreement of the dentists was assessed in 10 subjects (k > 0.91) for PlI and GI. For this purpose, we utilized the online Cohen Kappa calculator. The oral examination was conducted according to WHO criteria, which included the use of a dental mirror and a probe. Artificial light was used to ensure adequate visibility. For this procedure, patients were invited to a separate office that was previously prepared.

#### Assessment of the impact of stroke on patients’ functional status

The impact of stroke on the functional status of subjects in the study group was analyzed based on four clinical indicators, namely Addenbrooke’s Cognitive Examination Revised (ACE-R)^35,36^, Barthel Index (BI)^37^, The Functional Independence Measure (FIM)^38^, Sitting Balance Scale (SBS)^39,40^. Their brief descriptions are presented in Table 3.

**Table 3:** Clinical indicators used to evaluate the functional status of patients.

ACE-*R*	BI	FIM	SBS
Cognitive status of patients	Ability to perform basic daily activities independently.	Degree of assistance needed by the patient in performing daily life activities.	Postural balance while sitting.
The analysis of 5 cognitive domains allows for a maximum score of 100 points. A higher score indicates better cognitive abilities of the patient.	The assessment of 10 items of varying weight allows for a maximum of 20 points, which is equivalent to complete independence.	The scale includes 18 activities divided into motor and cognitive categories. Each is scored from 1 to 7 points. The analysis of the results indicates the degree of patients’ dependency in performing tasks. Scoring 120–126 points indicates full independence.	In 11 situations, scored from 0 to 4, the patient is assessed for the ability to maintain balance. A higher score indicates better balance of the subject.

ACE-R, Addenbrooke’s cognitive examination revised; BI, Barthel index; FIM, functional independence measure; SBS, sitting balance scale.

### Statistics

For statistical analysis, the GraphPad Prism 10 (GraphPad Software, La Jolla, CA, USA) and Past 4.13 (Øyvind Hammer) software were used. The level of statistical significance was set at *p* < 0.05. The distribution of the results was verified using the Kolmogorov-Smirnov test. In order to compare the differences between two-groups with a large number of variables, a multivariate permutation test was performed. In cases of abnormal distribution, the Mann-Whitney U test was used for comparisons between two groups. Correlations between biomarkers were assessed using Spearman rank-order correlation coefficient while the diagnostic utility of salivary chemokines and growth factors was analyzed using the ROC (Receiver Operating Characteristic) curve. For each parameter, the area under the curve (AUC) and optimal cutoff values were determined to ensure high sensitivity and specificity. For each group, the minimum number of participants was 20. The ClinCalc, an online calculator, was used to determine the minimum number of individuals needed for the study groups. The power in the statistical test was set at 0.8 (α = 0.05).

## Results

The clinical characteristics of the study group are provided in the supplement (Table S1).

### Chemokines

Chemokines constitute a large family of low molecular weight cytokines^41^. There are two types of chemokines: homeostatic (CTACK/CCL27, SDF-1α/CXCL12) and inflammatory (MCP-1/CCL2, MIP-1α/CCL3, MIP-1β/ CCL4, RANTES/CCL5, Eotaxin/CCL11, GRO-α/ CXCL1, IL-8/CXCL8, MIG/CXCL9, IP-10/CXCL10 and MIF)^42,43^. The latter are produced by activated leukocytes, and their production is induced by pro-inflammatory cytokines^43^. The common and fundamental function of both groups is the migration of responsive cells to specific locations, which, in the case of chemotactic cytokines, directs them to the inflamed tissues^42^.

Significantly higher levels of CTACK/CCL27, IL-8/CXCL8, MIG/CXCL9, MIF were noted in the unstimulated saliva of the study group compared to the control group (*p* ≤ 0.0001; *p* = 0.0023; *p* ≤ 0.0001, *p* = 0.0036, respectively). The levels of MCP-3/CCL7, eotaxin/CCL11 and IP-10/CXCL10 were, however, significantly lower in the unstimulated saliva of the study group compared to the control group (*p* ≤ 0.0001; *p* ≤ 0.0001; *p* = 0.0002, respectively).

**Fig. 2:**
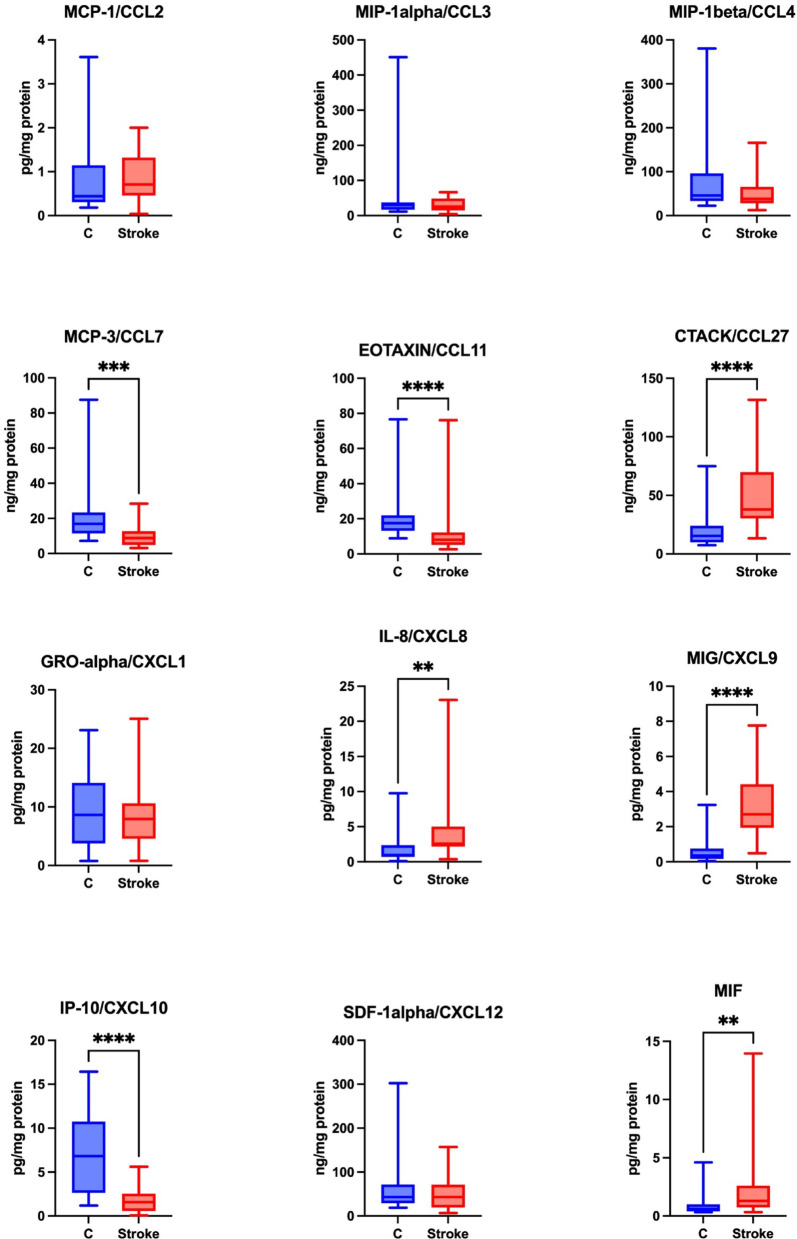
Concentration of chemokines in non-stimulated saliva of stroke patients compared to controls. MCP-1/CCL2: monocyte chemoattractant protein-1/chemokine ligands 2; MIP-1α/CCL3: macrophage inflammatory protein 1alpha/chemokine ligands 3; MIP-1β/ CCL4: macrophage inflammatory protein 1beta/chemokine ligands 4; MCP-3/CCL7: monocyte chemoattractant protein-3/chemokine ligands 7; eotaxin/CCL11: eotaxin/chemokine ligand 11; CTACK/CCL27: cutaneous T-cell-attracting chemokine/chemokine ligands 27; GRO-α/ CXCL1: growth-regulated alpha protein/chemokine (C-X-C motif) ligand 1; IL-8/CXCL8: interleukin 8/chemokine (C-X-C motif) ligand 8; MIG/CXCL9: monokine induced by gamma interferon/chemokine (C-X-C motif) ligand 9; IP-10/CXCL10: interferon gamma-induced protein 10/chemokine (C-X-C motif) ligand 10; SDF-1alpha/CXCL12: stromal cell-derived factor 1/chemokine (C-X-C motif) ligand 12; MIF: macrophage migration inhibitory factor; **p < 0.01; ***p < 0.001; ****p < 0.0001.

No statistically significant differences were found between the study and control groups regarding the levels of MCP-1/CCL2, MIP-1α/CCL3, MIP-1β/CCL4, GRO-α/CXCL1and SDF-1α/CXCL12 in unstimulated saliva (Fig. 2).

The concentration of RANTES in saliva was below the level of detection.

### Growth factors

Growth factors are responsible for cell differentiation and proliferation, and exhibit anti-inflammatory and anti-apoptotic effects, which improve stroke outcomes^14^. A statistically significant higher level of basic FGF, G-CSF, HGF, LIF, VEGF was noted in the unstimulated saliva of the study group compared to the control group (*p* ≤ 0.0001; *p* ≤ 0.0001; *p* ≤ 0.0001; *p* ≤ 0.0001; *p* = 0.0142, respectively). A statistically significant lower level of IL-3/MCGF and PDGF-BB was found in the unstimulated saliva of the study group compared to the control group (*p* ≤ 0.0001; *p* ≤ 0.0001, respectively).

**Fig. 3:**
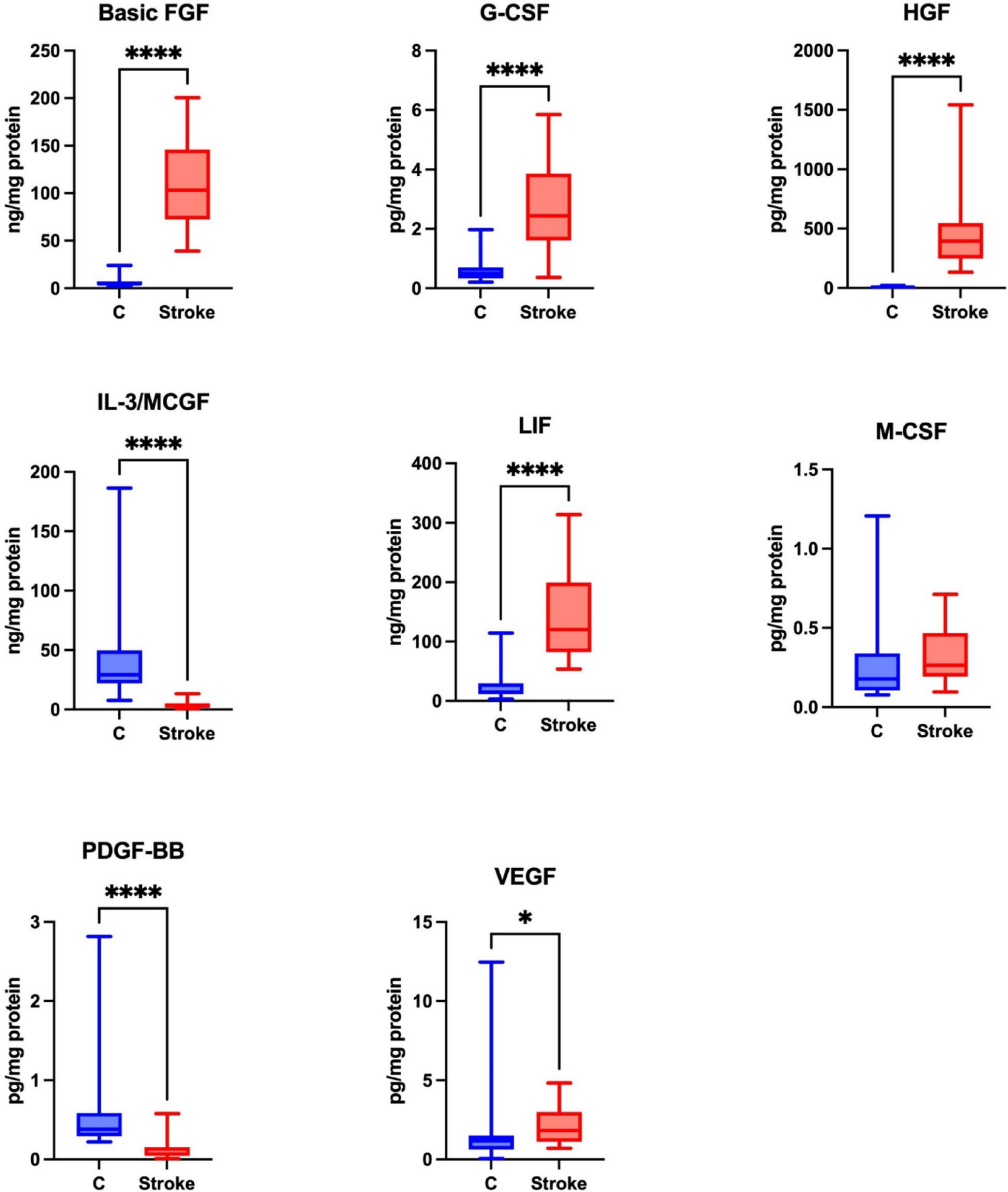
Concentration of growth factors in non-stimulated saliva of stroke patients compared to controls. Basic FGF: fibroblast growth factor; G-CSF: granulocyte colony-stimulating factor, HGF: hepatocyte growth factor; IL-3/MCGF: interleukin-3/mast cell growth factor; LIF: leukemia inhibitory factor; M-CSF: macrophage colony-stimulating factor, PDGF-BB; platelet-derived growth factor isoform BB; VEGF: vascular endothelial growth factor; *p < 0.05; ****p < 0.0001.

No statistical differences were observed regarding the level of M-CSF in the unstimulated saliva of the study and control groups (*p* = 0.0845) (Fig. 3).

The levels of GM-CSF, SCGF-β, SCF and NGF-β in saliva were undetectable.

### Correlations

**Fig. 4:**
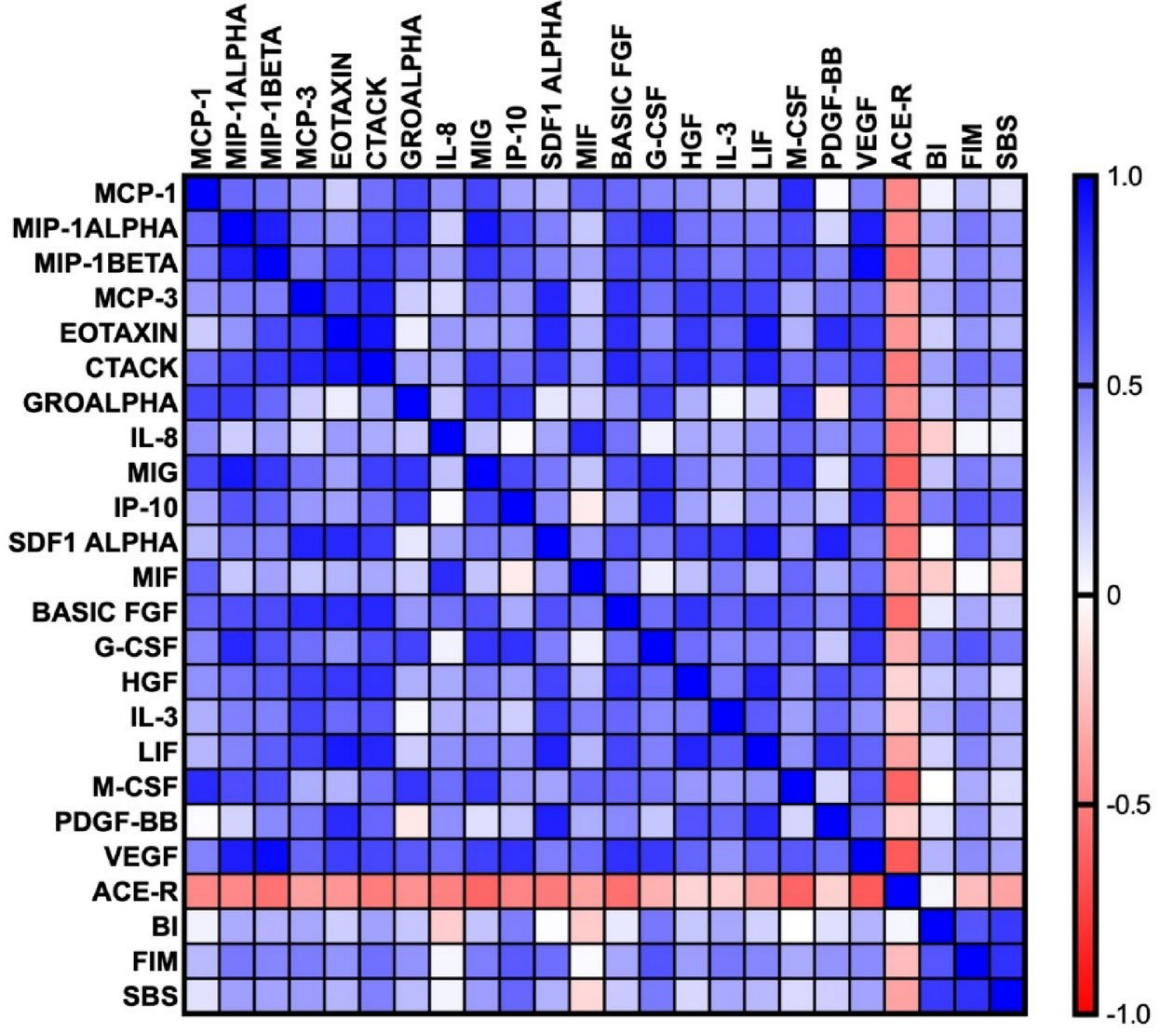
Correlations between the studied biomarkers. MCP-1/CCL2: monocyte chemoattractant protein-1/chemokine ligands 2; MIP-1α/CCL3: macrophage inflammatory protein 1alpha/chemokine ligands 3; MIP-1β/CCL4: macrophage inflammatory protein 1beta/chemokine ligands 4; MCP-3/CCL7: monocyte chemoattractant protein-3/chemokine ligands 7; eotaxin/CCL11: eotaxin/chemokine ligand 11; CTACK/CCL27: cutaneous T-cell-attracting chemokine/chemokine ligands 27; GRO-α/CXCL1: growth-regulated alpha protein/chemokine (C-X-C motif) ligand 1; IL-8/CXCL8: interleukin 8/chemokine (C-X-C motif) ligand 8; MIG/CXCL9: monokine induced by gamma interferon/chemokine (C-X-C motif) ligand 9; IP-10/CXCL10: interferon gamma-induced protein 10/chemokine (C-X-C motif) ligand 10; SDF-1α/CXCL12: stromal cell-derived factor 1/chemokine (C-X-C motif) ligand 12; MIF: macrophage migration inhibitory factor; basic FGF: fibroblast growth factor; G-CSF: granulocyte colony-stimulating factor; HGF: hepatocyte growth factor; IL-3/MCGF: interleukin-3/mast cell growth factor; LIF: leukemia inhibitory factor; M-CSF: macrophage colony-stimulating factor; PDGF-BB; platelet-derived growth factor isoform BB; VEGF: vascular endothelial growth factor; ACE-R: Addenbrooke’s Cognitive Examination Revised; BI: Barthel Index; FIM: The Functional Independence Measure; SBS: Sitting Balance Scale.

The correlations between inflammatory biomarkers and the clinical status of stroke patients are shown in Fig. 4.

We assessed the relationship between chemokines, growth factors and the functional status of patients. MCP-1/CCL2 was negatively correlated with ACE-R (*p* = 0.03, *r*=-0.46).

Similarly, MIP-1α/CCL3 and MIP-1β/CCL4 exhibited negative correlations with ACE-R (*p* = 0.03, *r*=-0.46; *p* = 0.01, *r*=-0.55, respectively). However, both were positively correlated with FIM (*p* = 0.01, *r* = 0.52; *p* = 0.03, *r* = 0.47, respectively).

We identified a positive correlation between MCP-3/CCL7 and FIM (*p* = 0.03, *r* = 0.51). CTACK/CCL27 was inversely correlated with ACE-R (*p* = 0.02, *r*=-0.51) but positively associated with FIM (*p* = 0.01, *r* = 0.55) and SBS (*p* = 0.03, *r* = 0.49).

Likewise, IL-8/CXCL8 demonstrated a negative correlation with ACE-R (*p* = 0.02, *r*=-0.49). MIG/CXCL9 exhibited a negative correlation with ACE-R (*p* = 0.003, *r*=-0.6), but a positive correlation with FIM (*p* = 0.02, *r* = 0.51).

A negative correlation was observed between IP-10/CXCL10 and ACE-R (*p* = 0.02, *r*=-0.49). Furthermore, IP-10/CXCL10 showed a positive correlation with BI (*p* = 0.02, *r* = 0.5), FIM (*p* = 0.001, *r* = 0.65) and SBS (*p* = 0.003, *r* = 0.595).

SDF-1α/CXCL12 was negatively correlated with ACE-R (*p* = 0.04 *r*=-0.52) and positively correlated with FIM (*p* = 0.02, *r* = 0.56).

We identified a negative correlation between basic FGF and ACE-R (*p* = 0.01, *r*=-0.56). G-CSF exhibited positive correlations with BI (*p* = 0.01, *r* = 0.53), FIM (*p* = 0.001, *r* = 0.67) and SBS (*p* = 0.01, *r* = 0.52). There was a positive correlation between IL-3/MCGF and FIM (*p* = 0.02, *r* = 0.53). Likewise, a positive correlation was observed between LIF and FIM (*p* = 0.03, *r* = 0.47). M-CSF was negatively correlated with ACE-R (*p* = 0.003, *r*=-0.61). Similarly, VEGF was inversely correlated with ACE-R (*p* = 0.01, *r*=-0.65). Finally, BI was positively correlated with FIM (*p* = 0.001, *r* = 0.66).

### ROC analysis

We used the ROC (Receiver Operating Characteristic) curve to assess the diagnostic utility of chemokines and growth factors, which illustrates the relationship between sensitivity and specificity and allows the determination of the optimal cutoff point between the study groups. In this study, the levels of growth factors and chemokines in saliva remarkably distinguished stroke patients from the control group. Significant diagnostic utility was particularly observed for growth factors, such as basic FGF, HGF, IL-3/MCGF and LIF (AUC = 1, sensitivity and specificity = 100%)(Table 4).

**Table 4:** Receiver operating characteristic (ROC) analysis for salivary growth factors and chemokines.

Biomarker	Area	95% CI	Cut off point	Sensitivity%	95% CI	Specificity%	95% CI	Likelihood ratio
Chemokines								
MCP-1/CCL2	0.6095	0.4370–0.7820	> 0.5531	72.73	51.85– 86.85%	63.64	42.95–80.27%	2
MIP-1α/CCL3	0.5083	0.3291–0.6875	< 29.00	59.09	38.73–76.74%	59.09	38.73–76.74%	1.444
MIP-1β/ CCL4	0.6104	0.4401–0.7807	< 62.91	76.19	54.91–89.37%	45.45	26.92–65.34%	1.397
MCP-3/CCL7	0.8301	0.7042–0.9561	< 13.84	84.21	62.43–94.48%	72.73	51.85–86.85%	3.088
Eotaxin/CCL11	0.8788	0.7643–0.9933	< 13.07	85.71	65.36–95.02%	81.82	61.48–92.69%	4.714
CTACK/CCL27	0.8636	0.7514–0.9758	> 24.99	85.71	65.36–95.02%	77.27	56.56–89.88%	3.771
GRO-α/ CXCL1	0.5455	0.3697–0.7212	< 9.062	61.9	40.88–79.25%	50	30.72–69.28%	1.238
IL-8/CXCL8	0.7662	0.6159–0.9165	> 1.812	85.71	65.36–95.02%	68.18	47.32–83.64%	2.694
MIG/CXCL9	0.9236	0.8425–1.000	> 0.7355	95.45	78.20–99.77%	77.27	56.56–89.88%	4.2
IP-10/CXCL10	0.874	0.7727–0.9753	< 3.474	90.91	72.19–98.38%	68.18	47.32–83.64%	2.857
SDF-1α//CXCL12	0.5401	0.3480–0.7322	< 48.54	58.82	36.01–78.39%	45.45	26.92–65.34%	1.078
MIF	0.7521	0.6054–0.8987	> 0.7623	77.27	56.56–89.88%	63.64	42.95–80.27%	2.125
Growth factors								
Basic FGF	1	1.000–1.000	> 20.05	100	85.13–100.0%	95.45	78.20–99.77%	22
G-CSF	0.9504	0.8824–1.000	> 0.7525	95.45	78.20–99.77%	86.36	66.67–95.25%	7
HGF	1	1.000–1.000	> 13.62	100	85.13–100.0%	95.45	78.20–99.77%	22
IL-3/MCGF	0.9952	0.9825–1.000	< 14.86	100	83.18–100.0%	95.45	78.20–99.77%	22
LIF	0.9978	0.9907–1.000	> 43.85	100	84.54–100.0%	95.45	78.20–99.77%	22
M-CSF	0.6529	0.4860–0.8198	> 0.1963	77.27	56.56–89.88%	59.09	38.73–76.74%	1.889
PDGF-BB	0.9351	0.8535–1.000	< 0.3040	90.48	71.09–98.31%	72.73	51.85–86.85%	3.317
VEGF	0.7299	0.5698–0.8900	> 1.352	70.59	46.87–86.72%	68.18	47.32–83.64%	2.218

MCP-1/CCL2, monocyte chemoattractant protein-1/chemokine ligands 2; MIP-1α/CCL3, macrophage inflammatory protein 1alpha/chemokine ligands 3; MIP-1β/CCL4, macrophage inflammatory protein 1beta/chemokine ligands 4; MCP-3/CCL7, monocyte chemoattractant protein-3/chemokine ligands 7; Eotaxin/CCL11, eotaxin/chemokine ligand 11; CTACK/CCL27, cutaneous T-cell-attracting chemokine/chemokine ligands 27; GRO-α/CXCL1, growth-regulated alpha protein/chemokine (C-X-C motif) ligand 1; IL-8/CXCL8, interleukin 8/chemokine (C-X-C motif) ligand 8; MIG/CXCL9, monokine induced by gamma interferon/chemokine (C-X-C motif) ligand 9; IP-10/CXCL10, interferon gamma-induced protein 10/chemokine (C-X-C motif) ligand 10; SDF-1α/CXCL12, stromal cell-derived factor 1/chemokine (C-X-C motif) ligand 12; MIF, Macrophage migration inhibitory factor; Basic FGF, fibroblast growth factor; G-CSF, granulocyte colony-stimulating factor; HGF, hepatocyte growth factor; IL-3/MCGF, interleukin-3/mast cell growth factor; LIF, leukemia inhibitory factor; M-CSF, macrophage colony-stimulating factor; PDGF-BB, platelet-derived growth factor isoform BB; VEGF, vascular endothelial growth factor.

## Discussion

The inflammatory response in stroke is initiated by the release of DAMPs from degenerating neuronal and non-neuronal brain cells. DAMPs include the heat shock proteins, chromatin-associated high mobility group box 1 (HMGB1), histones, and the S100 family of calcium-binding proteins, while the nonprotein group is comprised of adenosine triphosphate (ATP), uric acid, RNA, DNA, hyaluronan and heparan sulfate^44,45^. DAMPs activate immunological defense mechanisms via pattern recognition receptors (PRRs)^46^. In response to cerebral ischemia, astrocytes release pro-inflammatory cytokines as well as colony stimulating factors, such as GM-CSF and M-CSF, which contribute to the activation of surrounding microglia and monocytes^47,48^. These cells interact with each other through cytokine complexes^48^.

The blood-brain barrier (BBB) is a physical barrier that separates the central nervous system from the circulatory system^49^. The endothelial cells of the cerebral vessels contain tight junctions (TJs), which are crucial for maintaining brain homeostasis and BBB’s low permeability^50^. However, during a stroke, inadequate blood supply leads to cellular morphological changes, such as disruptions in the structure of TJs, which diminish BBB integrity and result in an excessive release of neuronal molecules into the cerebrospinal fluid and peripheral blood^5^. Due to the abundant vascularization of the salivary glands, many blood biomarkers can pass into the saliva. Chemokines have a molecular weight between 7 and 14 kDa^51^. Growth factors form a more diverse group - the molecular weight of the smallest basic FGF variant is 18 kDa^52^, while the molecular weight of HGF is 82 kDa^53^. Nevertheless, these compounds can enter the saliva through passive diffusion or ultrafiltration^22,54^. Low-molecular-weight inflammatory biomarkers derived from serum can also diffuse into saliva via the gingival crevicular fluid^55,56^. Hence, saliva reflects the composition of blood and thus represents a promising diagnostic material^57^. Under physiological conditions, saliva production varies between 0.5 and 1.5 L per day, with approximately 90% of saliva secreted by the three major salivary glands, i.e., the paired parotid, submandibular, and sublingual glands^58^.

**Fig. 5:**
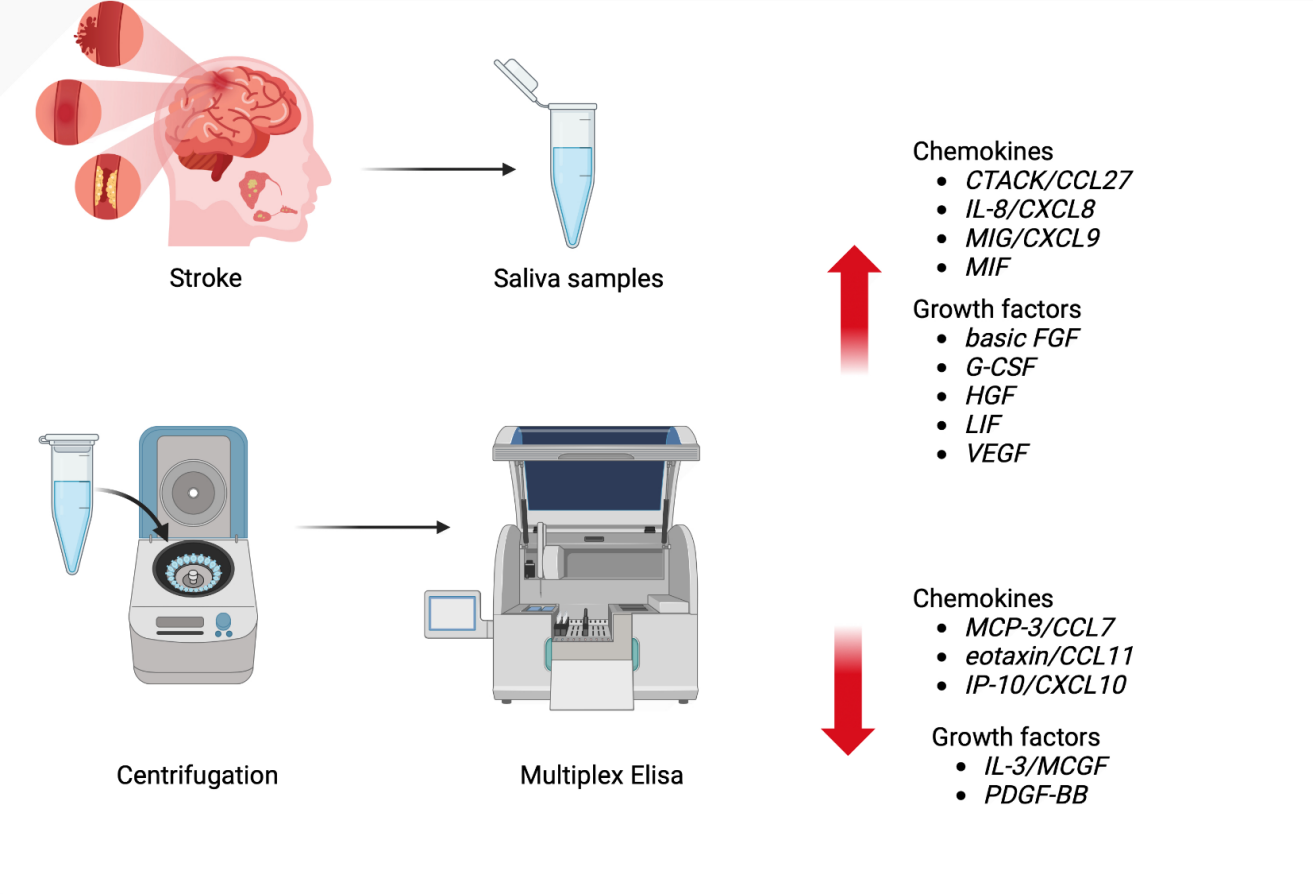
Graphical conclusions of the study (generated using canva.com and biorender.com). In the unstimulated saliva of stroke patients, a significantly higher content of chemotactic factors (CTACK/CCL27, IL-8/CXCL8, MIG/CXCL9, MIF) and growth factors (basic FGF, G-CSF, HGF, LIF, VEGF) was found compared to individuals from the control group. The content of MCP-3/CCL7, eotaxin/CCL11, IP-10/CXCL10, IL-3/MCGF and PDGF-BB was significantly lower in the saliva of patients from the study group. MCP-3/CCL7: monocyte chemoattractant protein-3/chemokine ligands 7; Eotaxin/CCL11: eotaxin/chemokine ligand 11; CTACK/CCL27: cutaneous T-cell-attracting chemokine/chemokine ligands 27; IL-8/CXCL8: interleukin 8/chemokine (C-X-C motif) ligand 8; MIG/CXCL9: monokine induced by gamma interferon/chemokine (C-X-C motif) ligand 9; IP-10/CXCL10: interferon gamma-induced protein 10/chemokine (C-X-C motif) ligand 10; MIF: Macrophage migration inhibitory factor; Basic FGF: fibroblast growth factor; G-CSF: granulocyte colony-stimulating factor, HGF: Hepatocyte growth factor; IL-3/MCGF: Interleukin-3/Mast cell growth factor; LIF: Leukemia inhibitory factor; PDGF-BB; platelet-derived growth factor isoform BB; VEGF: vascular endothelial growth factor.

In our study, the concentration of chemotactic factors (CTACK, IL-8, MIG, MIF) and growth factors (basic FGF, G-CSF, HGF, LIF, VEGF) was significantly higher in the unstimulated saliva of patients with ischemic stroke compared to the control group. In contrast, the content of MCP-3, eotaxin, IP-10, IL-3 and PDGF-BB was lower in the saliva of the study group (Fig. 5).

Chemokines are responsible for the directional migration and activation of leukocytes during inflammatory and homeostatic processes in stroke^51^. They are expressed in various brain cells (neurons, glial cells, and endothelial cells) as well as brain leukocytes and circulating blood leukocytes. The presence of leukocytes has also been demonstrated in saliva, where most of them enter the oral cavity through gingival crevices^59^. In our study, the content of CTACK and IL-8 was significantly higher in the saliva of individuals with ischemic stroke. CTACK is responsible for the recruitment of lymphocytes^60^, while IL-8 induces neutrophil chemotaxis^61^. Additionally, IL-8 stimulates immune cells to synthesize superoxide anions^62^, which can promote inflammation and edema in stroke^63^. The content of MIG and MIF was also significantly higher in the saliva of stroke patients. MIG’s function is to recruit T lymphocytes and differentiate effector T cells^64^, which confirms the involvement of chemokines in the development of the inflammatory response in stroke. The role of MIF in stroke remains controversial - on one hand, it can induce inflammation, while on the other, it inhibits neuronal apoptosis^65^. Interestingly, MIF can also induce the expression of brain-derived neurotrophic factor (BDNF), which exhibits neuroprotective effects^66,67^. Hence, chemokines may exhibit negative impact on brain tissue by inducing neuroinflammation and promoting leukocyte degranulation, nevertheless, their positive influence linked with stimulation of angiogenesis or angiostasis and cell survival should be also underlined^12,68,69^.

Elevated chemokine levels may be associated with a chronic condition leading to brain damage and a reduced likelihood of recovery^70–72^. Stroke affects various aspects of life, and accurately identifying the underlying causes can help reduce its negative effects. Post stroke cognitive impairments (PSCI) are especially common among stroke survivors. It is estimated that between 20%^73^ and 80%^74^ of individuals experience cognitive decline. PSCI encompasses a wide spectrum of cognitive impairments ranging from mild cognitive dysfunction to dementia^75^. It may manifest as problems with memory, concentration, executive functions or apraxia^76,77^. Cognitive impairment leads to an increased level of dependency and social withdrawal thus reducing the quality of life, as well as increasing the risk of depression^78–80^. Inflammation is a factor that is greatly responsible for the clinical consequences after stroke^81^. The knowledge of specific chemokines can identify individuals at risk for worse outcomes after stroke, and improve prognosis and treatment options. In this study, we found that several chemokines including CTACK (*p* = 0.02, *r*=-0.51), IL-8 (*p* = 0.02, *r*=-0.49) and MIG (*p* = 0.003, *r*=-0.6) were inversely correlated with cognitive function measured in ACE-R scale. ACE-R is a comprehensive clinical test designed to evaluate a wide range of cognitive impairments using 26 tasks, which are categorized into 5 subgroups. The domains assessed have assigned values - both memory and language count for 26 points each, verbal fluency for 14 points, attention and orientation for 18 points and visuospatial skills for 16 points^35^. Therefore, ACE-R scores provide valuable data taking into account both cognitive functions and cognitive deficits that patients exhibit after stroke^82^. The results presented here pave the way for further exploration into the long-term assessment of patients’ functional status after a stroke.

Angiogenesis is an adaptive mechanism in response to stroke^83^. This process includes both the formation of new blood vessels and the remodeling of the vascular system to create collateral vessels^84^. Angiogenesis is driven by growth factors, leading to an increase in capillary density within two weeks after stroke^85^. The appearance of vessels at the ischemic border enhances oxygen and nutrient supply to the affected region^86^. VEGF is considered the most important factor in angiogenesis due to its ability to recruit immune cells^87–89^. Basic FGF activates the caveolin-1/VEGF pathway and thus indirectly influences angiogenesis^90^. Moreover, basic FGF is a potent neurotrophic factor. It has been shown to improve BBB integrity by increasing the expression of tight junction proteins^91^. Guo et al. demonstrated that basic FGF levels in plasma increased 48 h after stroke, peaked on the third day and remained elevated until the 14th day after stroke^92^. In our study, the content of VEGF, basic FGF, G-CSF and HGF was significantly higher in the unstimulated saliva of patients with ischemic stroke compared to the controls. It is well known that G-CSF promotes the polarization of M2 microglial cells corresponding to an anti-inflammatory phenotype^93^. Indeed, G-CSF increases the expression of interleukin 10 (IL-10)^94^ and exhibits anti-apoptotic effects due to the activation of the PI3K/Akt pathway and increased synthesis of Bcl-XL (an anti-apoptotic protein in this pathway)^95^. HGF is considered a biomarker of endothelial damage^96^. In patients with atherothrombotic stroke, HGF levels in the blood were significantly higher compared to patients with cardioembolic stroke^20^. Neuroprotective properties are also attributed to LIF, which stimulates neuron differentiation from precursor cells^97^. Tian et al. observed a reduction in the extent of the ischemic area and an increase in regenerative processes in neurons exposed to LIF^98^. In our study, LIF levels in saliva were significantly higher in patients with ischemic stroke. Slevin et al. reported significantly lower LIF levels in the blood of stroke patients, with simultaneously increased expression in peri-infarct brain regions^99^. Interestingly, numerous studies have shown that for many biomarkers, there is a stronger correlation between their levels in the brain and saliva than between the brain and blood. This is due to the specific vascularization of the salivary glands and highlights the promising prospects of using saliva in the diagnosis of nervous system diseases^100–102^.

However, the content of eotaxin, IP-10 and MCP-3 was significantly lower in the saliva of the study group, which may indicate a weakening of the inflammatory response over time after stroke. Eotaxin has chemotactic activity towards eosinophils^103^. It is suggested that eotaxin may disrupt BBB continuity as it reduces the expression of tight junction proteins and promotes oxidative stress^104^. The actions of IP-10 and MCP-3 also include the migration of T lymphocytes and monocytes to the inflammatory site^105^. In this study, IP-10 and MCP-3 were associated with the physical status of stroke patients. Indeed, IP-10 was correlated positively with independence in daily activities (BI, *p* = 0.02, *r* = 0.5), the level of support needed in daily tasks (FIM, *p* = 0.001, *r* = 0.65) and the ability to maintain a stable sitting posture (SBS, *p* = 0.003, *r* = 0.595). MCP-3 levels were also correlated positively with the level of support needed in daily tasks (FIM, *p* = 0.03, *r* = 0.51). These correlations may indicate a beneficial effect of IP-10 and MCP-3 on the physical functioning of patients in the subacute phase of stroke. The BI (Barthel Index) scale identifies 10 items that correspond to various everyday activities, along with several statements assigned to each. The ten domains include feeding, grooming, bathing, dressing, bowel and bladder control, toilet use, stability while sitting, mobility, and ability to climb stairs, with a total of 20 points^106^. Thus, the BI scale helps to identify the most significant physical disabilities, facilitating targeted management of stroke-related consequences^107^. FIM (Functional Independence Measure) consists of 18 items, 13 functional areas are focused on physical status, whilst 5 items are related to cognition^108^. The motor-related tasks measure sphincter control, self-care, locomotion, and mobility, whereas cognitive ones evaluate a patient’s communication and social cognition^109^. These domains determine the severity of impairment, recognize the specific areas of decline and thus direct the path of rehabilitation^108^. SBS measures balance performance by assessing the ability to maintain upright position during sitting. It includes 11 functional tasks, each scored from 0 to 4 points, with higher scores indicating better performance^39^. Motor impairment in stroke patients refers to a limitation or loss of muscle function, movement, control or a reduced mobility^110^. It can manifest as abnormal muscle tone, reflected as spasticity or weakness, along with reduced dexterity or sensation^111,112^. Motor impairment following stroke occurs in approximately 80% of patients^110^.

Nevertheless, more than 30% of individuals remain chronically disabled^113^. Like cognitive dysfunction, motor impairment in stroke patients diminishes their ability to perform daily activities, lowers their quality of life, hinders their return to work, and places a significant socioeconomic burden on them^114^. Such aspects as community life and social roles, including work, family or education are also affected^115^. Inflammation is not only linked to cognitive decline but it also contributes to motor dysfunction by damaging neural connections involved in motor control^116^.

The levels of salivary growth factors (IL-3, PDGF-BB) were also lower in the saliva of stroke patients. IL-3 induces the proliferation of multipotent hematopoietic stem cells, neutrophils, eosinophils, megakaryocytes, macrophages, lymphoid cells, and erythroid cells^117^. It regulates the immune response by inducing the synthesis of IL-1 (interleukin 1), IL-6 (interleukin 6) and TNF-α (tumor necrosis factor alpha) by macrophages^118^. PDGF-BB increases the expression of MCP-3 in perivascular precursor cells and leads to increased macrophage accumulation^119^.

The increasing incidence of strokes necessitates improvements in diagnostics, enabling faster and non-invasive detection of the disease. An ideal stroke biomarker should distinguish between stroke and other neurological disorders, thereby reducing the number of false positive and false negative results^120^. Additionally, a stroke biomarker should be measurable by a validated analytical method, and its detection should be easy, fast, and inexpensive^121^. Due to its numerous advantages, saliva is garnering growing interest as a promising diagnostic material. Unfortunately, the available literature lacks studies evaluating the usefulness of salivary chemokines and growth factors in the diagnosis/monitoring of disease progression in stroke patients. In our previous study we showed that salivary TNF-α distinguished stroke patients from healthy individuals with high specificity and sensitivity. TNF-α acts as a biomarker to distinguish patients with normal cognition and those with mild to moderate cognitive dysfunction in the study group^122^. In this study, we demonstrated that the concentrations of basic FGF, HGF, IL-3, and LIF most effectively differentiate ischemic stroke patients from the control group (AUC = 1, sensitivity and specificity = 100%). The diagnostic potential of biomarkers can also be confirmed by statistically significant correlations with the cognitive and functional status of patients. We found that the concentration of basic FGF negatively correlates with patients’ cognitive functions on the ACE-R scale (*p* = 0.01, *r*=-0.56), and the salivary levels of IL-3 and LIF positively correlate with scores on the FIM scale (*p* = 0.02, *r* = 0.53; *p* = 0.03, *r* = 0.47, respectively). However, it is important to remember that the assessment of chemokine and growth factor profiles in extracellular fluids (saliva, blood, cerebrospinal fluid) provides only limited information about inflammatory processes occurring in brain tissue. Furthermore, based on the results of our study, it is difficult to draw conclusions about the clinical usefulness of salivary chemokines and growth factors in stroke diagnostics. We did not measure the concentration of inflammatory mediators immediately after the onset of stroke symptoms. Although the number of patients has been statistically determined, further studies on a larger population are necessary to determine reference values and to compare ischemic to hemorrhagic stroke. However, our study shows the usefulness of saliva for the non-invasive assessment of chemokines and growth factors in patients with ischemic stroke. Multiplex tests can be a useful tool for identifying inflammatory mediators in saliva. Of the 25 biomarkers evaluated, only GM-CSF, SCGF-β, SCF, NGF-β and RANTES were below the level of detection, preventing us from fully assessing the chemokine and growth factor profile in the saliva of stroke patients. Indeed, the concentration of many molecules in saliva is lower when compared to blood, which makes it impossible to use this diagnostic material to assess all biomarkers^123^. Nevertheless, the ease of collection, non-invasiveness, and non-infectious nature of saliva samples are advantages for using this biofluid in laboratory medicine^124^. Saliva collection techniques reduce patient anxiety and discomfort and allow for the collection of multiple samples throughout the day without the involvement of medical personnel^25^. There are also no contraindications for collecting saliva from individuals with coagulation disorders^125^. Therefore, additional multicenter studies are needed to evaluate the relationship between the concentration of inflammatory biomarkers, the location of vascular changes, the extent of brain damage, and the duration of ischemia and hypoxia. Moreover, an ideal stroke biomarker should appear in saliva shortly after the onset of the disease.

In summary, alterations in chemokine and growth factor levels in saliva may suggest an inflammatory etiology of ischemic stroke. Saliva can be used for the non-invasive assessment of inflammatory mediators in stroke patients. Inflammation may be related to the cognitive and functional status of patients. Further research is needed to explain the differences in salivary profiles of inflammatory mediators in stroke, evaluate the diagnostic utility of chemokines and growth factors in clinical practice.

## Electronic supplementary material

Below is the link to the electronic supplementary material.

Supplementary Material 1.

## Data Availability

The data that support the findings of this study are available from the corresponding author upon reasonable request.
