# Supplementary material for: Salivary chemokines and growth factors in patients with ischemic stroke
Source: Sci Rep. 2025 Apr 12;15:12676. doi: 10.1038/s41598-025-97974-5 (PMC11993640; doi:10.1038/s41598-025-97974-5)
Supplement: Supplementary file 1 — Supplementary Material 1. [file 41598_2025_97974_MOESM1_ESM.docx]

| **Characteristics** | | **C (n =22)** | | | **Stroke (n =22)** | | | **P-value** |
| --- | --- | --- | --- | --- | --- | --- | --- | --- |
|  |  | *25% Percentile* | *Median* | *75% Percentile* | *25% Percentile* | *Median* | *75% Percentile* |  |
| Age | | 60 | 65 | 71.25 | 60 | 65 | 71.25 | > 0.9999 |
| Sex | male n (%) | 13 (59%) | | | 13 (59%) | | | > 0.9999 |
|  | female n (%) | 9 (41%) | | | 9 (41%) | | |  |
| Education | primary n (%) | 2 (9%) | | | 1 (4.5%) | | | > 0.9999 |
|  | vocational n (%) | 8 (36.5%) | | | 9 (41%) | | |  |
|  | secondary n (%) | 8 (36.5%) | | | 11 (50%) | | |  |
|  | university n (%) | 4 (18%) | | | 1 (4.5%) | | |  |
| Place of residence | urban centre n (%) | 5 (22.5%) | | | 7 (32%) | | | > 0.9999 |
|  | small town n (%) | 8 (36.5%) | | | 10 (45.5%) | | |  |
|  | rural area or small village n (%) | 9 (41%) | | | 5 (22.5%) | | |  |
| Houshold member(s) | with family member n (%) | 20 (91%) | | | 21 (95.5%) | | | > 0.9999 |
|  | none n (%) | 2 (9%) | | | 1 (4.5%) | | |  |
| Comorbidities | hypertension n (%) | 12 (54.5%) | | | 11 (50%) | | | > 0.9999 |
|  | type 1 diabetes n (%) | 0 | | | 0 | | |  |
|  | type 2 diabetes n (%) | 9 (41%) | | | 8 (36.5%) | | |  |
|  | thyroid diseases n (%) | 2 (9%) | | | 2 (9%) | | |  |
|  | epilepsy n (%) | 0 | | | 0 | | |  |
|  | atrial fibrillation n (%) | 4 (18%) | | | 5 (22.5%) | | |  |
|  | limb thrombosis n (%) | 4 (18%) | | | 6 (27%) | | |  |
| Drugs | < 5 drugs/day n (%) | 19 (86.5%) | | | 17 (77.5%) | | | > 0.9999 |
|  | > 5 drugs/day n (%) | 3 (13.5%) | | | 5 (22.5%) | | |  |
| *Dental examination* | | | | | | | | |
| PlI | | 1 | 1.5 | 2 | 1 | 2 | 2 | 0.501 |
| GI | | 0.75 | 2 | 2 | 0 | 1 | 2 | 0.1956 |
| DMFT | | 21.5 | 32 | 32 | 23.25 | 30.5 | 32 | 0.7604 |
| *Cognitive and physical functional status* | | | | | | | | |
| ACE-R | | ND | | | 67.75 | 73 | 81.5 | ND |
| BI | | ND | | | 3.5 | 9.5 | 12 | ND |
| FIM | | ND | | | 28.25 | 62 | 87.5 | ND |
| SBS | | ND | | | 5.75 | 10.5 | 14.25 | ND |

Table S1. General characteristics of both groups of stroke patients and controls.

ACE-R: Addenbrooke’s Cognitive Examination Revised; BI: Barthel Index; DMFT: The Decayed, Missing and Filled Teeth; GI: Gingival Index; FIM: The Functional Independence Measure; PlI: Plaque Index; SBS: Sitting Balance Scale.
